# Cognitive pathways to communicative-pragmatic dysfunction in schizophrenia: The role of autistic symptoms^☆^

**DOI:** 10.1016/j.scog.2026.100449

**Published:** 2026-06-16

**Authors:** G. Agostoni, M.F. D'Incalci, M. Bechi, J. Sapienza, M. Spangaro, V. Cirulli, M. Buonocore, F. Martini, F. Frau, C. Barattieri di San Pietro, L. Bischetti, C. Guglielmino, F. Cocchi, R. Cavallaro, V. Bambini, M. Bosia

**Affiliations:** a Schizophrenia Research and Clinical Unit, IRCCS San Raffaele Scientific Institute, Milan, Italy; b School of Medicine, Vita-Salute San Raffaele University, Milan, Italy; c Laboratory of Neurolinguistics and Experimental Pragmatics (NEP), Department of Humanities and Life Sciences, University School for Advanced Studies IUSS, Pavia, Italy

**Keywords:** Psychosis, Autism Spectrum Disorder, Language, Theory of Mind, Executive functions

## Abstract

**Background:**

Communicative-pragmatic dysfunction is recognized as a core feature of schizophrenia, as well as a hallmark of Autism Spectrum Disorders (ASD), strongly intertwined with neurocognitive and sociocognitive domains. Autistic symptoms, i.e., autistic-like traits below ASD diagnostic threshold, occur in over 50% of patients with schizophrenia, affecting social cognition and functioning. Strikingly, their specific link with pragmatics remains unexplored. This study tests the relationship between autistic symptoms and pragmatics in schizophrenia, modelling their interplay with executive functions (EF) and Theory of Mind (ToM), in the context of the sprout model of pragmatics.

**Methods:**

A sample of 125 individuals with schizophrenia was assessed for autistic symptoms, pragmatics, ToM, and EF. Path analysis examined direct and indirect effects of autistic symptoms on global pragmatics and on pragmatic production and comprehension, with EF and ToM as mediating variables.

**Results:**

Autistic symptoms were negatively associated with EF, ToM, and pragmatics. Path analysis revealed that autistic symptoms have a direct effect on global pragmatics, as well as an indirect one, via EF and ToM consecutively. Furthermore, autistic symptoms indirectly affected both pragmatic production, via EF, and comprehension, through EF and ToM sequentially.

**Conclusions:**

Results show a strong link between autistic symptoms and pragmatics in schizophrenia, highlighting a cognitive pathway from EF to ToM in supporting pragmatics, in line with the sprout model, with a key role of the former in production and of the latter in comprehension. Clinically, this study highlights that assessing autistic symptoms in routine care may help identify patients at higher risk for pragmatic deficits and guide targeted rehabilitation strategies.

## 1 Introduction

Pragmatic competence is defined as the ability to use language appropriately and flexibly across different kinds of communicative contexts (Domaneschi and Bambini, 2020; Bambini and Lecce, 2025). Impairment in the domain of pragmatics has been recognized as a core feature of schizophrenia, affecting up to 80% of patients (Colle et al., 2013; Bambini et al., 2016; Kalandadze et al., 2019), with substantial negative impact on daily functioning and quality of life (Agostoni et al., 2021). In particular, difficulties with context-dependent uses of language have been documented since earlier studies, including work from the late 20th century (e.g., Chapman, 1960; Chaika, 1982; Andreasen and Grove, 1986; Drury et al., 1998), and have been consistently confirmed by more recent research (e.g., Champagne-Lavau and Stip, 2010; Bambini et al., 2016; Parola et al., 2018), highlighting persistent weaknesses in organizing discourse and interpreting expressions such as metaphors, irony, and indirect speech. Also, pragmatics is typically disrupted in people with Autism Spectrum Disorder (ASD), encompassing a range of difficulty that goes from conversational skills to non-literal language understanding (Loukusa and Moilanen, 2009; Kalandadze et al., 2019; Eigsti et al., 2025). Noteworthy, autistic traits, which are autistic-like symptoms that do not reach the diagnostic threshold for ASD, have been found to be a significant clinical feature within the schizophrenia spectrum, with a great impact on functional outcome (Barlati et al., 2019; Bechi et al., 2020b). This scenario unveils potential overlaps between the spectra of ASD and schizophrenia on the one hand, and pragmatic impairment on the other. However, to date no previous studies have yet examined the specific interactions between autistic symptoms and pragmatic abilities in individuals with schizophrenia. In the present study, we address this gap by investigating how these dimensions mechanistically contribute to pragmatic disruption, in the context of the sprout model of pragmatics (Frau et al., 2024).

Previous research has linked pragmatic deficits to other key alterations typically observed in schizophrenia, such as impairments in cognitive domains, particularly executive functions (EF) and sociocognitive abilities, such as Theory of Mind (ToM). However, in the last few decades, the way these domains interact along the causal pathways underlying pragmatic impairment has been a matter of debate (Champagne-Lavau and Stip, 2010; Bambini et al., 2016; Parola et al., 2018; Parola et al., 2020; Agostoni et al., 2023). In line with post-Gricean theories conceptualizing language as intrinsically intertwined with mind reading abilities (Mazzarella and Noveck, 2021), several studies have emphasized the central role of social cognition (Bambini and Lecce, 2025), with some even proposing pragmatics as reducible to a submodule within ToM abilities (Sperber and Wilson, 2002). Conversely, other studies have proposed that pragmatic competence reflects a broader metacognitive process, inherently dependent on higher-order EF even in the absence of ToM involvement (Kissine, 2016; Kissine, 2021). More recently, a comprehensive view of the cognitive underpinnings of pragmatic impairments has emerged, recognizing the interplay between social and cognitive domains (Hyter, 2017; Snow and Douglas, 2017; Bosco et al., 2018; Domaneschi and Bambini, 2020). A recent meta-analysis directly addressed this issue, confirming that pragmatic competence is associated with both ToM and EF in patients with schizophrenia (Frau et al., 2024). Specifically, a stronger relation with pragmatics was found for social cognition compared to EF, and the former was shown to mediate the association between EF and pragmatic competence (Frau et al., 2024). Based on these findings, the authors proposed a sprout model in which pragmatic abilities stem from social cognition, which serves as a superordinate cognitive domain involved in processing social communicative intentions, while EF constitute a more general cognitive substrate, supporting both social cognition and pragmatic competence (Frau et al., 2024).

In parallel, additional clinical and psychopathological factors may modulate the relationships between EF, ToM and pragmatics (Bambini et al., 2020; Bambini et al., 2022a; Frau et al., 2024). Among these, autistic symptoms deserve particular attention for several reasons. First, although schizophrenia and ASD are two distinct diagnostic categories (American Psychiatric Association, 2022), several overlapping areas between the autistic and schizophrenia spectra are reported (King and Lord, 2011; Hallerbäck et al., 2012; Matsuo et al., 2015; Barlati et al., 2016). Indeed, studies highlighted that a subgroup of patients with schizophrenia also show autistic symptoms, including difficulties in social interactions, emotional processing, communicative abilities, and motor abnormalities. The prevalence of these symptoms in schizophrenia ranges from 10 to 61%, while the prevalence for a co-diagnosis of both schizophrenia and ASD ranges from less than 1 to 52% of patients (King and Lord, 2011; Kästner et al., 2015; Kincaid et al., 2017; Barlati et al., 2019). Moreover, studies reported that genetic, biological, clinical, cognitive and functional characteristics may be shared between ASD and schizophrenia, suggesting possible common defects in neurodevelopmental pathways (Burbach and van der Zwaag, 2009; Hommer and Swedo, 2015; Chisholm et al., 2015; Kincaid et al., 2017; Kushima et al., 2018; De Crescenzo et al., 2019).

Second, while autism is characterized by tremendous heterogeneity in cognitive abilities, pragmatic language deficits are generally considered to be universal, and are observed even in individuals with otherwise age-appropriate cognitive and linguistic abilities (Paul, 2007; Loukusa and Moilanen, 2009; Eigsti et al., 2011; Eigsti et al., 2025). Autistic individuals struggle with multiple aspects of pragmatics (Loveland et al., 1988), including comprehension of language (Åsberg, 2010), comprehending indirect requests (Paul and Cohen, 1985; Rajendran et al., 2005), back-channeling (Irvine et al., 2016), and figurative language (Kalandadze et al., 2019). Interestingly, high levels of autistic symptoms in schizophrenia have been linked to poorer premorbid social and academic adjustment since childhood, suggesting that patients with greater autistic symptoms may undergo earlier disruptions in the development of social, cognitive and daily functioning, which may in turn exacerbate later impairments following the onset of schizophrenia (Bechi et al., 2020a). Consistently with this view, high levels of autistic symptoms have been associated also with more severe impairments in ToM and neurocognitive functioning (including EF), as well as poorer daily functioning, and weaker responses to rehabilitative interventions (Bechi et al., 2020a; Bechi et al., 2020b). This evidence has led to the hypothesis of a possible “double dose” of impairment resulting from the co-occurrence of autistic symptoms in schizophrenia (Vaskinn and Abu-Akel, 2019), with relevant implications for clinical, neurocognitive, and functional outcomes.

Despite the growing body of evidence independently linking autistic symptoms, EF, and ToM to pragmatic abilities, no study to date has investigated how these dimensions interact with each other in schizophrenia, nor how their interplay may differentially affect distinct components of pragmatics, such as production and comprehension. Addressing this gap may provide a more fine-grained understanding of the mechanisms underlying communicative dysfunction, moving beyond the identification of isolated deficits toward the characterization of their functional organization.

The present study aimed to comprehensively examine the interplay between autistic symptoms, ToM, EF, and pragmatic abilities using a path analysis approach. We hypothesized: (i) that autistic symptoms could be integrated, along with EF and ToM, in a model predicting pragmatic competence in schizophrenia; (ii) in line with the sprout model (Frau et al., 2024), that pragmatic competence is closely associated with both ToM and EF in schizophrenia, with possible differences when considering different pragmatic facets; and (iii) that autistic symptoms could affect pragmatics both directly and indirectly by in turn affecting EF and ToM abilities.

By integrating these domains into a single model, this study can advance our current understanding of pragmatic competence and its cognitive and clinical determinants in schizophrenia. Disentangling the complex interrelations among these domains may also provide theoretical insights into the cognitive architecture of pragmatic competence, while at the same time highlighting, at the clinical level, predictors of pragmatic abilities that could inform the design of personalized and more effective rehabilitation strategies.

## 2 Methods

### 2.1 Participants

We recruited one hundred and twenty-five patients with schizophrenia (DSM-5-TR criteria, American Psychiatric Association, 2022) at IRCCS San Raffaele Scientific Institute, Milan, Italy. Inclusion criteria were: age between 18 and 65, being native Italian speakers, being on antipsychotic therapy, and being able to provide written informed consent. Exclusion criteria were: severe traumatic brain injury, neurological disorders, intellectual disability, alcohol or substance abuse in the preceding 6 months, severe psychotic exacerbations in the preceding 3 months. The protocol was approved by the local Ethical Committee, following the principles of the Declaration of Helsinki. All subjects provided informed consent.

### 2.2 Assessment

Patients were assessed for the severity of autistic symptoms by trained psychiatrists. Trained psychologists evaluated pragmatic abilities, ToM, and EF.

*Autistic symptoms* were evaluated with the PANSS Autism Severity Score (PAUSS; Kästner et al., 2015), which is the only validated measure for the assessment of autistic symptoms in schizophrenia. Three scores, representing the three symptom domains of autism, are derived from specific PANSS items: 1) “Difficulties in Social Interaction” score (items 1 ‘blunted affect’, 3 ‘poor rapport’, and 4 ‘social withdrawal’ from the Negative Scale); 2) “Difficulties in Communication” score (items 5 ‘difficulties in abstract thinking’ and 6 ‘lack of spontaneity and flow of conversation’ from the Negative Scale); and 3) “Stereotypies/Narrowed Interests” score (item 5 ‘mannerism’ and 15 ‘preoccupation’ from the General Scale, and item 7 ‘stereotyped thinking’ from the Negative Scale). The PAUSS Total score is the sum of the subscales. Higher PAUSS scores represent a higher severity of the autistic symptoms. The measure of interest was PAUSS Total score.

*Pragmatics* was evaluated with the Assessment of Pragmatic Abilities and Cognitive Substrates test (APACS; Arcara and Bambini, 2016), a validated tool to assess pragmatic production and comprehension skills in Italian through six tasks: Interview (a speech production task); Description (a description task); Narratives (a narrative comprehension task); Figurative Language 1 (a multiple-choice comprehension task with idioms, metaphors, and proverbs); Humor (a multiple-choice humor comprehension task); and Figurative Language 2 (a verbal explanation task with idioms, metaphors, and proverbs). In addition to a score for each task, the APACS provides three composite scores: the Pragmatic Production score, the Pragmatic Comprehension score, and the APACS Total score (calculated from Pragmatic Production and Comprehension scores). The measures of interest were: APACS Production, Comprehension, and Total scores.

*ToM* was assessed using the ToM Picture Sequencing Task (PST; Brüne, 2003). The PST consists of six cartoon stories depicting different scenarios. In each story, four cards are presented in mixed order, and participants are first asked to order the cards in a logical sequence of events (Sequencing task) and then to answer questions on characters' mental states, motivations, and beliefs (Questionnaire task). The Questionnaire includes first to third order false beliefs questions, queries involving the understanding of cheating detection, and two reality questions, included to rule out major attentional problems. From Total Sequencing and Total Questionnaire scores, we derive the PST Total score, as global measure of ToM abilities. The measure of interest was PST Total score.

*EF* were evaluated using the Brief Assessment of Cognition in Schizophrenia (BACS; Keefe et al., 2004; Anselmetti et al., 2008), evaluating the cognitive domains usually impaired in schizophrenia. The variable of interest was BACS Executive functions score (Tower of London subtest).

### 2.3 Statistical analysis

First, we used a Pearson's correlation matrix to analyze the correlation between pragmatics and predictor variables of interest, namely autistic symptoms (PAUSS Total score), ToM (PST Total score) and EF (BACS Tower of London score), including also age, education and duration of illness as possible influencing factors. This procedure was run to select predictors to include in the subsequent analysis; *p*-value was corrected according to Bonferroni's correction for multiple comparisons (*p* < .005).

Then, we performed path analysis, a special case of structural equation modelling, to test the hypothesized direct and indirect effects of predictors on pragmatics. Specifically, two path analysis models were run: the first path analysis evaluated the direct and indirect effects of predictors on global pragmatics, while the second, more complex, path analysis evaluated the direct and indirect effects of predictors on both pragmatic production and comprehension. In both path analysis models, autistic symptoms scores were entered as exogenous variables (which directly affect both endogenous variables and dependent variables, and indirectly affect dependent variables via endogenous variables), ToM and EF were included as intervening endogenous variables (which directly affect dependent variables), while pragmatics (global pragmatics in the first model, and both pragmatic production and comprehension in the second model) was entered as dependent variable. To obtain the most consistent models, a robust selection criterion of the predictors and the paths to include in the analysis was applied, based on the correlation matrix (*p* < .005). Regarding sample size, it ‘should exceed 100 observations regardless of other data characteristics to avoid problematic solutions and obtain acceptable fit concurrently’ (Nasser and Wisenbaker, 2003). Our sample complies with the recommendation of 10 to 20 cases per parameter (Kline, 2011), and good fit can be reached despite having a sample size of less than 200 (Hayduk et al., 2007; Markland, 2007).

The model fit of both models was examined by several goodness-of-fit indexes: χ^2^, χ^2^ test *p*-value, the comparative fit index (CFI), the standardized root mean square residual (SRMR), the root mean square error of approximation (RMSEA), and Tucker-Lewis Index (TLI). Models are considered to have a good fit when the χ^2^ statistic is non-significant, CFI is greater than 0.90, SRMR and RMSEA are 0.08 or less (Hu and Bentler, 1999). However, when the sample size is small and the models have few degrees of freedom, the RMSEA too often falsely indicates a poor fit (Hu and Bentler, 1999; Kenny et al., 2015).

To test the statistical significance of the hypothesized mediations in the model, bias-corrected confidence intervals (95%) were calculated through the bootstrapping procedure (5000 samples), as recommended by Shrout and Bolger (2002).

Data analysis were conducted in R (R Core Team, 2022).

## 3 Results

### 3.1 Sample description

The sample was composed of 125 participants (77 males and 48 females), with a mean age of 38.46 years (SD = 12.76), mean education of 12.33 years (SD = 2.84), mean age at onset of 23.47 years (SD = 6.57), and a mean duration of illness of 16.14 years (SD = 10.89).

Table 1 shows mean scores for autistic symptoms, pragmatics, ToM and executive functions performance.

**Table 1:** Autistic symptoms, pragmatics, ToM, and executive functions performance.

	Mean (SD)	Range
*PAUSS*		
Difficulties in social interaction score	9.19 (2.67)	4–17
Difficulties in communication score	5.87 (2.01)	2–11
Stereotypies/narrowed interests score	8.68 (2.08)	4–15
Total score	23.75 (5.43)	10–37
*APACS*		
Pragmatic production	0.89 (0.07)	0.46–1
Pragmatic comprehension	0.74 (0.15)	0.21–0.93
APACS total score	0.81 (0.10)	0.38–0.96
*BACS*		
Executive functions	13.78 (4.40)	3–22
*PST*		
PST total score	45.26 (11.57)	0–59

*Note.* Data are given as mean and standard deviation (SD), and as minimum and maximum score. PAUSS = PANSS Autism Severity Score; APACS = Assessment of Pragmatic Abilities and Cognitive Substrates test; BACS = Brief Assessment of Cognition in Schizophrenia; PST = Picture Sequencing Task.

### 3.2 Correlation matrix

**Fig. 1:**
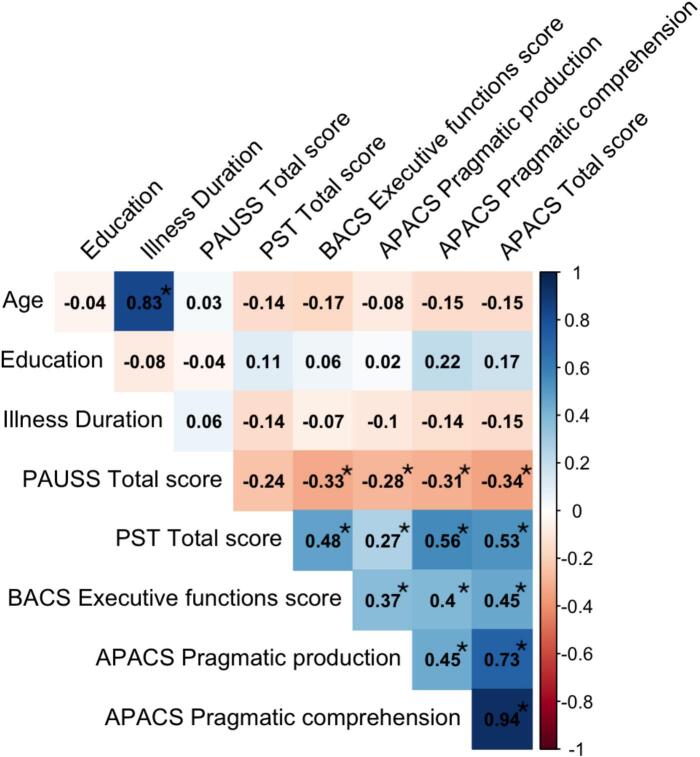
Correlation matrix. *Note*. The figure shows the correlation matrix between demographical, clinical, pragmatic, sociocognitive, and cognitive data. * represent significant correlations after Bonferroni's correction for multiple comparisons (*p* < .005).

To evaluate patterns of correlations, as well as to select variables to include in the following analysis, a Pearson correlation matrix was run between demographic, clinical, pragmatic, sociocognitive, and cognitive data. The *p*-value was corrected according to Bonferroni's correction for multiple comparisons (*p* < .005). Results showed significant correlations between: 1) PAUSS Total score and BACS EF score (*r* = −0.33, *p* < .001), APACS Pragmatic production (*r* = −0.28, *p* = .001), APACS Pragmatic comprehension (*r* = −0.31, *p* = .001), and APACS Total score (*r* = −0.34, *p* < .001); 2) BACS EF score and PST Total score (*r* = 0.48, *p* < .001), APACS Pragmatic Production (*r* = 0.37, *p* < .001), APACS Pragmatic Comprehension (*r* = 0.40, *p* < .001), and APACS Total score (*r* = 0.45, *p* < .001); and 3) PST Total score and APACS Pragmatic production (*r* = 0.27, *p* = .002), APACS Pragmatic comprehension (*r* = 0.56, *p* < .001), and APACS Total score (*r* = 0.53, *p* < .001). No significant correlations were found with age, education, or illness duration after Bonferroni correction. Results are shown in Fig. 1.

These results further supported our a-priori hypothesis to include autistic symptoms, EF and ToM as predictors of pragmatic abilities. Moreover, we selected the paths to test in the model based on the significant correlations emerged between autistic symptoms, EF and ToM. This criterion was adopted to ensure model parsimony and theoretical consistency, including only variables that were empirically and conceptually linked, while minimizing model complexity and the risk of overfitting.

### 3.3 Path analysis on global pragmatics

A first path analysis model was performed to examine the direct and indirect effects of autistic symptoms, evaluated with PAUSS total score, on global pragmatics, evaluated via APACS total score, through EF and ToM, respectively evaluated by BACS EF score and PST Total score.

**Fig. 2:**
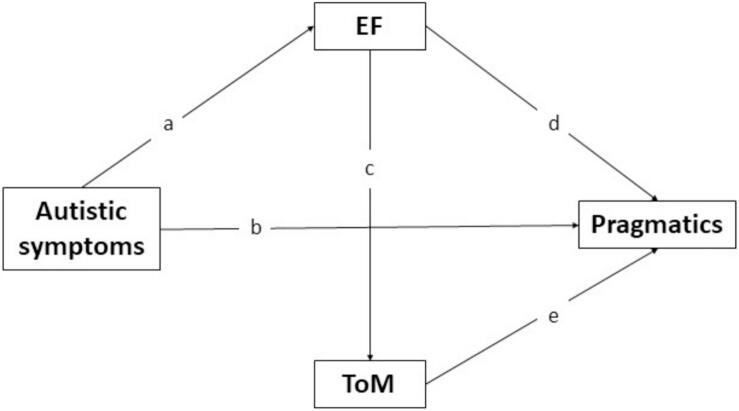
Path analysis on global pragmatics.

The model was significant and explained 35% of the variance in global pragmatics, and showed good fit indices: χ^2^(1) = 1.36, *p* = .24, CFI = 0.996, TLI = 0.978, RMSEA = 0.05 (90% CI [0.00, 0.25]), SRMR = 0.03. Moreover, the model explained 11% of the variance in BACS EF score and 23% in PST Total score. Path model is shown in Fig. 2.

As for direct effects, higher PAUSS score was significantly associated with poorer BACS EF score (path a; β = −0.27, *p* < .001), which in turn predicted better PST Total score (path c; β = 1.26, *p* < .001). Moreover, APACS total score was directly predicted by PAUSS score (path b; β = −0.003, *p* = .04), BACS EF score (path d; β = 0.005, *p* = .04), and PST Total score (path e; β = 0.003, *p* = .005).

As for indirect effects, PAUSS score showed a significant indirect path on APACS total score consecutively via BACS EF score and PST Total score (path ace; β = −0.001, *p* = .04). Additionally, PAUSS score also showed an indirect effect on APACS total score through BACS EF score (path ad; β = −0.001, *p* = .061), which showed a trend of significance. Lastly, when all indirect paths were combined, the total indirect effect of PAUSS score on pragmatics was significant (β = −0.002, *p* = .009), yielding a significant total effect of −0.006 (*p* < .001).

### 3.4 Path analysis on pragmatic production and comprehension

A second path analysis model was performed to examine the direct and indirect effects of autistic symptoms, evaluated with PAUSS total score, on pragmatic production and comprehension, respectively evaluated via APACS Production and Comprehension scores, through EF and ToM, respectively evaluated by BACS EF score and PST Total score.

**Fig. 3:**
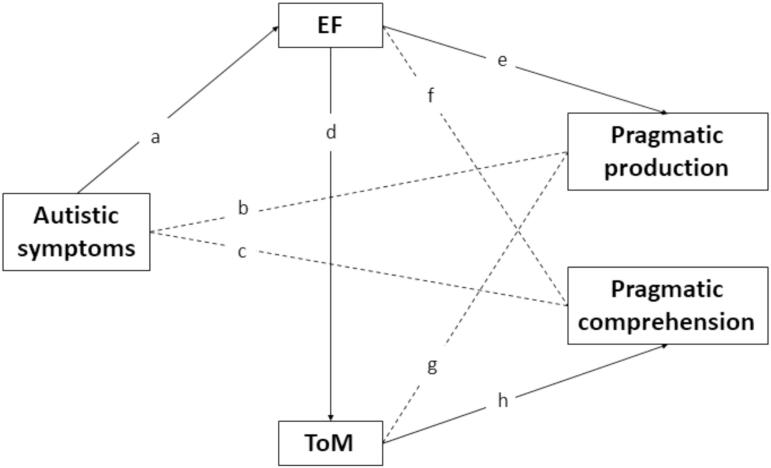
Path analysis on pragmatic production and comprehension. *Note*. Dotted path = nonsignificant path.

The model was significant, with 17% of the variance explained in APACS Production score and 34% in APACS Comprehension score, and showed excellent fit indices: χ^2^(1) = 1.36, *p* = .24, CFI = 0.997, TLI = 0.972, RMSEA = 0.05 (90% CI [0.00, 0.25]), SRMR = 0.02. As in model 1, also this model confirmed that 11% of the variance was explained for BACS EF score and 23% for PST Total score. Path model is shown in Fig. 3.

As for direct effects, higher PAUSS score was significantly associated with poorer BACS EF score (path a; β = −0.27, *p* < .001), which in turn predicted better PST Total score (path d; β = 1.26, *p* < .001). Regarding pragmatic production, APACS Production score was significantly predicted by BACS EF score (path e; β = 0.005, *p* = .02), while its associations with PAUSS score (path b; β = −0.002, *p* = .12) and PST Total score (path g; β = 0.001, *p* = .40) were not significant. As for pragmatic comprehension, APACS Comprehension score was significantly predicted by PST Total score (path h; β = 0.006, *p* = .002), while its association with PAUSS score showed a trend toward significance (path c; β = −0.004, *p* = .06), and the effect of BACS EF score was not significant (path f; β = 0.004, *p* = .23).

As for indirect effects, PAUSS score showed a significant indirect path on APACS Production score via BACS EF score (path ae; β = −0.001, *p* = .03). Furthermore, PAUSS score showed a significant indirect path on APACS Comprehension score via BACS EF and PST Total score (path adh; β = −0.002, *p* = .04). Lastly, when all indirect paths were combined, the total indirect effect of PAUSS score on pragmatic outcomes was significant (β = −0.005, *p* = .009), yielding a significant total effect (β = −0.010, *p* = .001).

## 4 Discussion

This study aimed to model the interplay between autistic symptoms and pragmatic abilities in schizophrenia, considering the contribution of EF and ToM, using a path analysis approach. To our knowledge, this is the first study providing an integrated approach that combines a clinically relevant dimension, namely autistic symptoms, and considerations of the cognitive architecture supporting pragmatics.

Pragmatic deficits are widespread in schizophrenia, with a significant impact on social and occupational functioning, autonomy, and quality of life (Agostoni et al., 2021). Pragmatics represents a complex ability relying on the integration of multiple cognitive domains, particularly neurocognitive and sociocognitive domains. Consistent with recent meta-analytic evidence (Frau et al., 2024), pragmatic competence appears to depend on both EF and ToM, with the latter playing a superordinate role that mediates the relationship between executive control and communicative-pragmatic processes. However, since these domains account for only a part of the variance in pragmatic performance, this leaves room for the identification of additional factors that may contribute to pragmatic impairment and that may interact within the broader communicative architecture. Thus, unravelling mechanisms underlying pragmatic impairment is crucial to characterize the clinical and cognitive architecture of pragmatics, as well as to identify targets for personalized rehabilitation strategies.

Recently, growing research has focused on autistic symptoms, as they are a meaningful but still under-assessed dimension within the schizophrenia spectrum (Kincaid et al., 2017; Barlati et al., 2019). In this view, autistic symptoms appear as a relevant clinical target, being highly prevalent in schizophrenia and associated with poorer prognosis, particularly affecting social cognition and social functioning (De Crescenzo et al., 2019; Bechi et al., 2020b). Moreover, autistic symptoms include communicative difficulties that can be traced back to specific pragmatic dysfunctions. Surprisingly, while pragmatic disorder is systematically assessed in ASD, the interplay between autistic symptoms and pragmatic abilities has not yet been considered in schizophrenia. Our results offer evidence to address this gap.

First, correlation analysis confirmed the expected pattern of associations between pragmatic abilities and the key cognitive and clinical domains evaluated in this study. In detail, pragmatic measures were significantly related to autistic symptoms, EF, and ToM, in line with previous evidence (Bambini et al., 2016; Parola et al., 2020; Frau et al., 2024). Also, we found a significant relationship between higher autistic symptoms and both lower ToM and EF abilities, in line with the view of interconnected mechanisms rather than isolated deficits (Bechi et al., 2020b).

Moving to the path analysis, the first model confirmed that autistic symptoms directly and indirectly predict global pragmatic ability, with indirect effects consecutively mediated first by EF and then by ToM. Specifically, autistic symptoms were associated with executive performance, which in turn predicted ToM and, ultimately, pragmatic outcome. These findings support the hypothesis that autistic symptoms may negatively affect pragmatic competence both directly and through their impact on higher-order cognitive mechanisms, thus confirming evidence that links autistic traits to widespread neuro- and socio-cognitive dysfunctions in schizophrenia (Bechi et al., 2020b; Alvarez et al., 2023). This result corroborates the hypothesis of Vaskinn and Abu-Akel (2019) that the co-occurrence of schizophrenia and autistic symptoms is associated with a “double dose” of deficit in different neurocognitive domains, and extends such hypothesis to pragmatic impairment. Also, given the synergic role of EF and ToM, our results align with previous studies (Frau et al., 2024) that highlight the hierarchical role of sociocognitive and cognitive skills as determinants of pragmatics. Specifically, this model shows that EF influence both ToM and pragmatic abilities, while ToM has a stronger direct effect on pragmatics and also partially mediates the impact of EF. This pattern of association between variables further supports the pragmatic sprout hypothesis (Frau et al., 2024), according to which pragmatic competence emerges from the sequential integration of cognitive control and sociocognitive processes.

The second model further clarified these mechanisms by distinguishing between pragmatic production and comprehension. Here, EF specifically predicted pragmatic production, whereas both EF and ToM emerged as the key predictors of pragmatic comprehension, consistent with the view that these abilities provide complementary support for communication (Frau et al., 2024). Importantly, autistic symptoms showed significant indirect effects on both pragmatic domains: via EF for production and via both EF and ToM for comprehension, thus highlighting their crucial role in shaping communicative abilities. Interestingly, in the second model results do not show a direct effect of PAUSS on pragmatic abilities, in contrast with results on the pragmatic global model. We can hypothesize that the impact of autistic symptoms is distributed across both pragmatic domains and is captured when considering the overall pragmatic score, but is masked by the stronger effects of EF and ToM when pragmatic facets are analysed separately. This result may partially align with Eigsti et al. (2025), who reported that the link between ToM and pragmatics diminishes in remitted adults with ASD, together with an improvement in pragmatic performance, suggesting that the strength and patterns of interplay between different domains may vary according to the severity of deficits and symptoms. In this light, we can speculate that a more direct relationship between autistic symptoms and pragmatic comprehension might emerge in individuals with higher PAUSS levels, while this link could be weaker in people with milder autistic symptoms. Furthermore, our results also fit with the evidence that pragmatic production is less impaired than comprehension in schizophrenia, as reported by Frau et al. (2025). In particular, the deficit in production domain seems to affect mainly coherence and discourse organization, while other aspects of pragmatic discourse are relatively preserved (Meister et al., 2026).

Innovatively, our results show distinct cognitive pathways according to the pragmatic domain addressed, as we found different paths for pragmatic production and pragmatic comprehension. On the one hand, EF, along with autistic symptoms, confirmed to be the fertile ground supporting pragmatic production, highlighting the pivotal role of inhibitory processes in appropriately using and adapting language according to the context (Schettino et al., 2010; Li et al., 2017; Parola et al., 2020). On the other hand, we found a path starting with autistic symptoms, then affecting first EF and then ToM, with a final effect on pragmatic comprehension. This is in line with Frau et al. (2024), who showed a complex interplay between EF and ToM domains in determining pragmatics. Moreover, our findings are consistent with previous results (Frau et al., 2025) showing that pragmatic production is generally less affected than comprehension and, crucially, is not directly linked to ToM. This pattern was observed not only in schizophrenia but also across different clinical populations, suggesting that pragmatic comprehension relies more heavily on metantalizing skills, while production primarily depends on executive control mechanisms.

Taken together, these findings suggest that autistic symptoms contribute to pragmatic dysfunction in schizophrenia through both neurocognitive and sociocognitive pathways, supporting the view of a shared and partially overlapping substrate among these domains, with differences across expressive and receptive modalities. Moreover, they emphasize that assessing autistic symptoms in schizophrenia is clinically relevant, as they appear to be key predictors of abilities that are significantly impaired in schizophrenia, namely cognitive, sociocognitive and pragmatic skills.

Some limitations of this study should be acknowledged. First, the sample consisted of a relatively homogeneous group of clinically stable patients, which may limit the generalizability of the findings to more acute or functionally impaired populations. Second, the cross-sectional design prevents causal inference on the directionality of effects. Future longitudinal studies should clarify whether autistic symptoms precede or co-evolve with pragmatic and cognitive dysfunctions over the illness course. Moreover, future studies could integrate the present dimensional approach by comparing subgroups of patients with different levels of autistic symptoms. However, since autistic symptoms can be conceptualized as a continuum of severity within the schizophrenia spectrum and may exert graded effects on cognitive and functional outcomes, we adopted a dimensional approach as it could provide a more fine-grained understanding of the mechanisms underlying communicative dysfunction, which may be partially limited by categorical grouping. In addition, the application of established cutoffs (Kästner et al., 2015) to our sample resulted in highly unbalanced groups, highlighting both statistical and conceptual challenges in adopting a categorical approach. Finally, to disentangle these contributions and further clarify the specificity of the underlying mechanisms, future studies should consider more specific subcomponents of both autistic symptoms (e.g., difficulties in social interaction, difficulties in communication, and stereotypies) and pragmatic abilities (e.g., metaphor, irony, indirect speech). Indeed, different dimensions of autistic symptoms may show distinct associations with pragmatic impairment or reflect a common underlying vulnerability, and different components of pragmatics may differentially rely on executive and sociocognitive processes (Happé, 1993; Langdon et al., 2002; Andrés-Roqueta and Katsos, 2017).

In conclusion, from a clinical point of view this study provides novel evidence that autistic symptoms play a significant role in determining pragmatic impairments in schizophrenia, both directly and indirectly through their impact on executive and mentalizing mechanisms. These results underscore the importance of systematically assessing both autistic traits and communicative-pragmatic abilities in clinical evaluations. The assessment of autistic symptoms, which can be easily derived from routinely administered scales such as the PANSS, may represent a useful first step to identify patients who could benefit from further in-depth evaluations of neurocognitive, sociocognitive, and pragmatic domains. Furthermore, from a research point of view, our results support the literature on the importance of the synergic effects of EF and ToM on pragmatics, and add a piece to the puzzle by pointing out the differential effects when we account for different facets of pragmatic abilities. Accordingly, autistic symptoms might contribute to distinguishing different socio-communicative and linguistic phenotypes within the schizophrenia spectrum (Bambini et al., 2022a; Frau et al., 2025), thus acknowledging not only different manifestations of communicative-pragmatic difficulties but also distinct underlying cognitive and clinical mechanisms. Integrating this information can guide individualized rehabilitation programs orienting clinicians toward the most relevant targets for rehabilitative interventions. For instance, pragmatic-focused programs (e.g., PragmaCom; Bambini et al., 2022b) could be offered to people with autistic symptoms and possibly combined with other interventions addressing ToM-like aspects in people showing higher levels of autistic symptoms, to maximize benefits for patients' communicative abilities as well as social functioning and quality of life.

## CRediT authorship contribution statement

**G. Agostoni:** Writing – original draft, Visualization, Project administration, Methodology, Formal analysis, Data curation, Conceptualization. **M.F. D'Incalci:** Writing – original draft, Project administration, Methodology, Data curation. **M. Bechi:** Project administration, Methodology, Investigation, Data curation. **J. Sapienza:** Methodology, Formal analysis, Data curation. **M. Spangaro:** Data curation. **V. Cirulli:** Data curation. **M. Buonocore:** Data curation. **F. Martini:** Data curation. **F. Frau:** Methodology. **C. Barattieri di San Pietro:** Methodology. **L. Bischetti:** Methodology. **C. Guglielmino:** Data curation. **F. Cocchi:** Data curation. **R. Cavallaro:** Writing – review & editing, Supervision, Conceptualization. **V. Bambini:** Writing – review & editing, Supervision, Methodology, Funding acquisition, Conceptualization. **M. Bosia:** Writing – review & editing, Supervision, Methodology, Funding acquisition, Conceptualization.

## Fundings

The project was partly supported by the Italian Ministry of University and Research under Grant Number 2022289RNA (“The Fragility of Metaphors (FraMe): learning, loosing, and how to train them”) awarded to M.B. and V.B.

## Declaration of competing interest

None.
